# Health facility preparedness of maternal and neonatal health services: a survey in Jumla, Nepal

**DOI:** 10.1186/s12913-021-07054-3

**Published:** 2021-09-28

**Authors:** Pasang Tamang, Padam Simkhada, Paul Bissell, Edwin van Teijlingen, Rose Khatri, John Stephenson

**Affiliations:** 1 School of Human and Health Sciences, University of Huddersfield, Huddersfield, UK; 2 Global Health, School of Human and Health Sciences, University of Huddersfield, Huddersfield, UK; 3 Reproductive Health Research, Centre for Midwifery, Maternal & Perinatal Health, Bournemouth University, Poole, UK; 4 Public Health, Liverpool John Moores University, Liverpool, UK; 5 Biomedical Statistics, School of Human and Health Sciences, University of Huddersfield, Huddersfield, UK

**Keywords:** Health facilities, Health system, Maternal health, Neonatal health, Quality of care

## Abstract

**Background:**

Over the past 20 years, Nepal has seen major improvements in childhood and maternal survival. In 2015, the Nepalese government introduced a new federal political structure. It is unclear how this has affected the health system, and particularly, maternal and child health care. Hence, this study aims to describe and analyse health facility preparedness in the light of the federalization process with regards to providing appropriate and timely maternal and neonatal health services.

**Methods:**

A descriptive cross-sectional study was conducted in Jumla district, Nepal in 2019 covering all 31 state health facilities (HF) to assess the availability of maternal and neonatal health services including appropriate workforce and access to essential medicines. Tests of association between demographic factors and the probability of a facility experiencing a shortage of essential medicine within the last 3 months were also conducted as exploratory procedures.

**Results:**

Out ot 31 HFs, more than 90% of them had all their staff positions filled. Most facilities (*n* = 21) had experienced shortages of essential medicines within the past 3 months. The most common out of stock medicine were: Amoxicillin (*n* = 10); paracetamol (n = 10); Vitamin A (*n* = 7); and Metronidazole (*n* = 5). Twenty-two HFs had referred maternal and newborn cases to a higher centre within the past 12 months. However, more worryingly, twenty HFs or their catchment communities did not have emergency ambulance transport for women and newborns.

**Conclusion:**

HFs reported better staffing levels than levels of available drugs. HFs should be supported to meet required minimal standards such as availability of essential medicines and the provision of emergency ambulance transport for women and newborns.

## Background

Over the past 20 years, Nepal has seen major improvements in childhood and maternal survival. However, despite an increase in institutional delivery, maternal and neonatal mortality remains still high due to the poor quality of care during pregnancy, childbirth and the postpartum period [26]. Hence improving access to health care which is affordable and of appropriate quality during these periods can play a crucial role in the reduction of maternal and neonatal mortality [24]. According to the World Health Organization [26], high-quality care requires an appropriate infrastructure, motivated and skilled health workers and effective case management using evidence-based clinical practice. High quality maternal health services could prevent 1 million newborn deaths and half of the annual global maternal mortality [8].

Improving the quality of health services is a key strategy to achieve the United Nations (UN) Sustainable Development Goal 3 (SDG3) to be achieved by 2030 [16]. To achieve this aim, “quality health care must be safe, effective, timely, efficient, equitable and people-centered” [23]. Particularly relevant to the maternal and newborn health services are three SDG targets: 3.1 “reduce the global maternal mortality ratio (MMR) to less than 70 per 100,000 live births”; 3.2 “end preventable deaths of newborns and children under 5 years of age, with all countries aiming to reduce neonatal mortality to at least as low as 12 per 1,000 live births”; and 3.8 “Achieve universal health coverage (UHC), including financial risk protection, access to quality essential health-care services and access to safe, effective, quality and affordable essential medicines and vaccines for all” [20]. However, if the MMR is not reducing on average by 7.5% each year between now and 2030, this SDG target will not be achieved. Low- and middle-income countries (LMIC), in particular, face considerable challenges in providing quality, affordable and universally accessible maternal and neo-natal care [10].

### Maternal and neonatal health in Nepal

Improving maternal and neonatal health is a national health priority in Nepal; yet every year around 1200 women die during pregnancy or while giving birth, mostly in rural areas [19]. Moreover, every year an estimated 23,000 children die before turning five; and three out of five infants who die do so within 28 days of birth [2]. Many of these deaths could be prevented with better maternal and child health services closer to home [6].

**Fig. 1:**
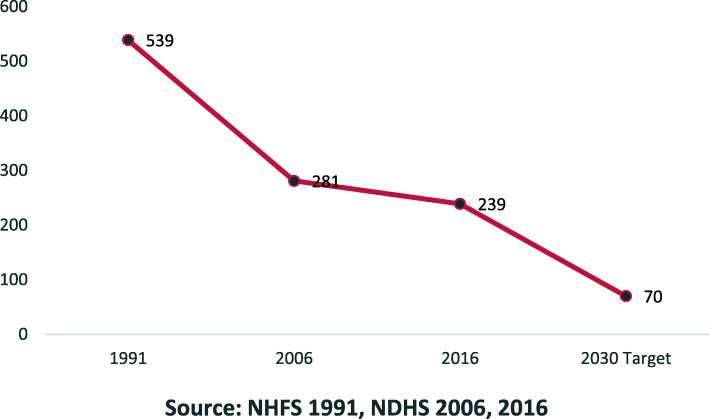
Trends in Maternal Mortality Ratio (MMR)

MMR reduced by 44% worldwide between 1990 and 2015 [25]; however, in Nepal, MMR reduction has been slow (Fig. 1). At its current pace Nepal’s MMR will only reduce by 59 deaths per 100,000 live births to 180 deaths per 100,000 live births by 2030, which is about 2.5 times higher than the SDGs target. Whilst in Jumla district which lies in Karnali province, 44.3% of pregnant women had four ANC (Antenatal Care) checkups, as recommended by the WHO, in the fiscal year 2017/2018, this is less than the provincial (Karnali) average (54.9%) or the national average (49.8%) [13]. The proportion of women having institutional delivery in Jumla (56.4%) was also lower than the provincial (Karnali) average (67.3%) [13]. Furthermore, only 20.7% of women in the Jumla district had three post-natal care (PNC) check-ups as recommended; which should take place within 24 h, 72 h and 7 days of delivery.

**Fig. 2:**
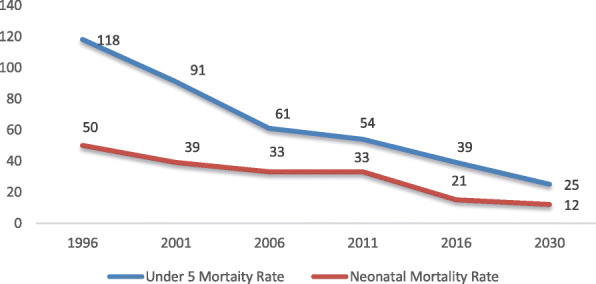
Trends in under −5 and Neonatal Mortality Rate (Source: NHFS 1991, 2001, NDHS 2006, 2011, 2016)

The Neonatal Mortality Rate (NMR) accounts for nearly half the total of under 5 years mortality rate in Nepal. The recent Nepal Multi Indicator Cluster Survey (NMICS) showed some reduction in the NMR to 15 per 1000 live births (Fig. 2) [12]. Improvements in maternal and child survival, however, could be challenged by changes in the political system and the transition in Nepal to a federal state.

### Federalization in Nepal

**Fig. 3:**
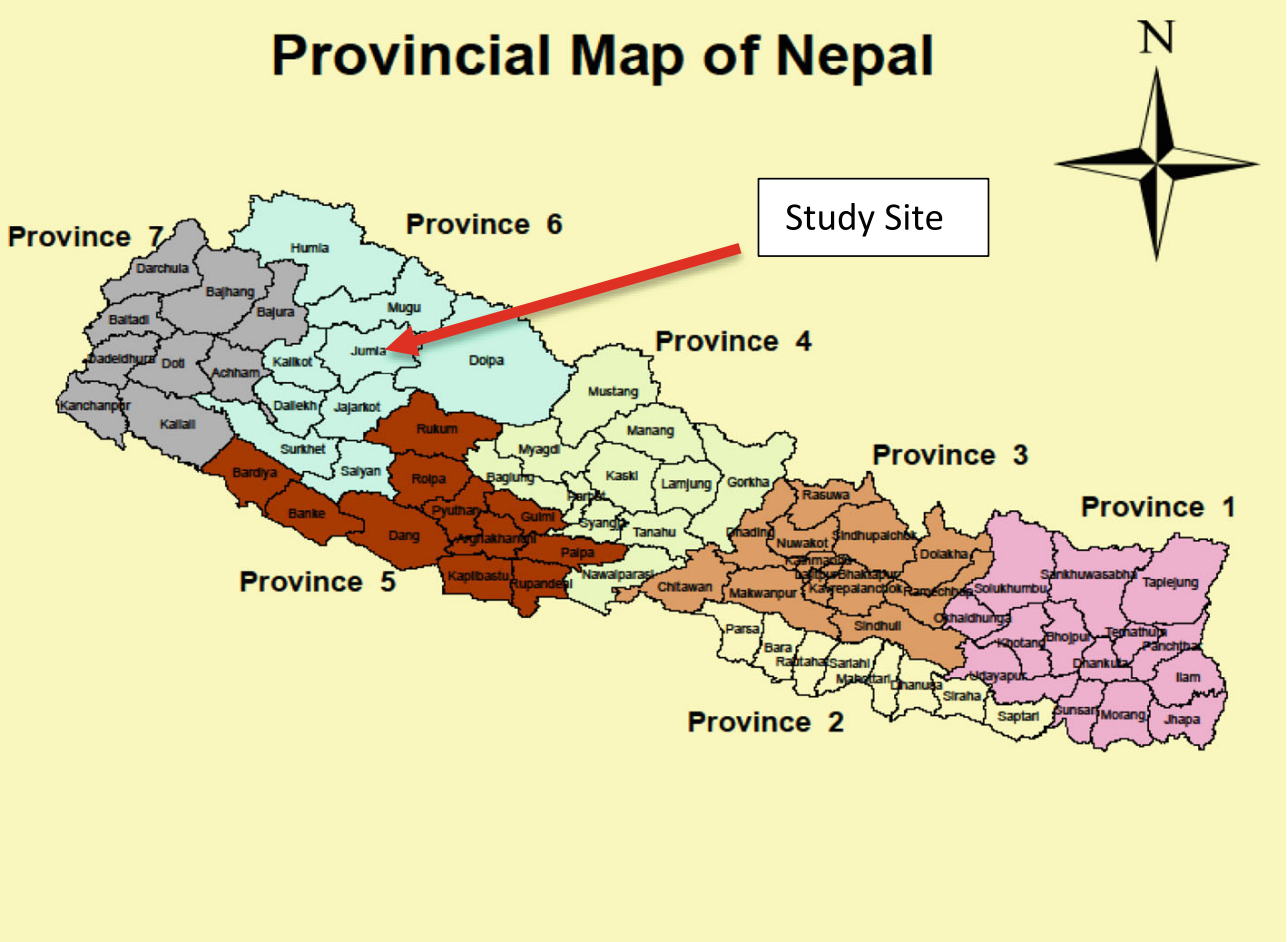
Map of Nepal after Federalisation: seven provinces and 77 districts (showing study site). The figure was generated using a Geographic Information System (GIS)

Nepal’s political system has seen significant changes over the past three decades. After a decade-long Maoist rebellion, a popular democratic movement brought about a change in the political system in 2006. An interim constitution was promulgated, and the king abdicated. This resulted in the new 2015 Constitution, when Nepal became a federal republic. As part of federalization, Nepal has been divided into three autonomous governance levels: federal/national, provincial and local. Each level has its own powers to make policy, generate and utilize resources locally [15]. There are seven provinces, 77 districts and 753 local units in Nepal (Fig. 3).

Prior to federalization, the Ministry of Health and Population (MoHP) provided resources and services to meet the health needs of the population. Under the new political structure, health services have been decentralised with the transfer of power and responsibility for planning, administration and decision-making from central government to local authorities [17]. However, there is no clear line of authority nor communication between the local, provincial or national health levels within the system. Moreover, local and provincial authorities must determine their own priorities and resource planning, which may compromise national targets to reduce MMR and NNMR [17]. It is unclear as to what impact this is having on the health system and particularly on maternal and child health care. Hence, this study aims to describe and analyse health facility preparedness in terms of availability of human resources, essential medicine and adequate equipment, in relation to providing appropriate and timely maternal and neonatal health services in light of federalization.

This article covers the methods section which consists of the information of the study design, methods of data collection, participant recruitments and study site followed by findings of the study, discussion and the conclusion.

## Methods

**Fig. 4:**
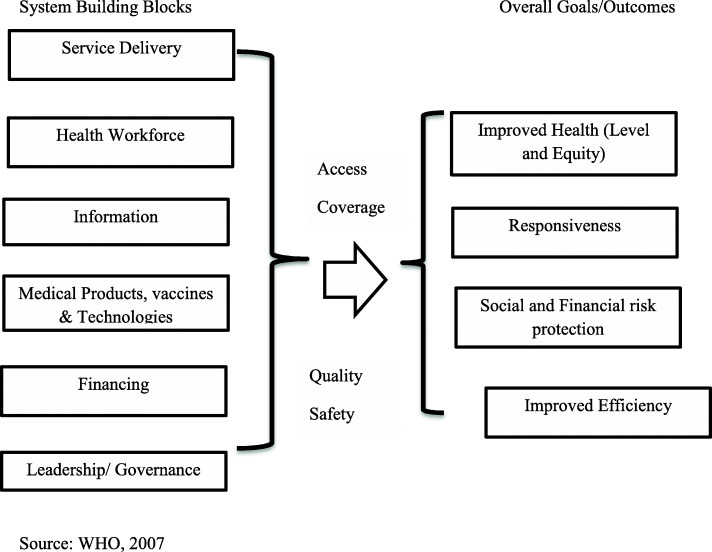
The WHO Health System Framework

**Fig. 5:**
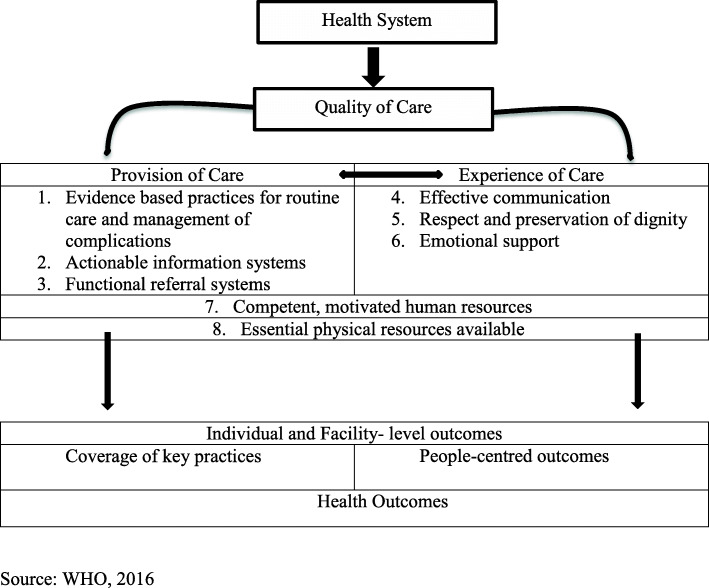
WHO framework for the quality of maternal and newborn health care

A descriptive cross-sectional study was conducted in the Jumla district of Nepal in 2019. A health-facility (HF) survey was conducted in all 31 state-run HFs (29 Health Posts, one primary Health Care Centre and one hospital) in Jumla district. A census method was applied using a survey questionnaire guided by the WHO Health System Framework [22] (Fig. 4) and the WHO framework for the quality of maternal and newborn healthcare [26] (Fig. 5). These frameworks were chosen as they are critical to health system improvement and achievement of core SDG 3 targets. Previous research has indicated that the WHO Health System Framework helps to assess the status of the health facilities [11].

The questionnaire contained information relating to the provision of care and facility readiness such as health service delivery, maternal and newborn care services, infrastructure such as water and electricity, availability of skilled health workers, access to essential medicines and a health information system since they are a critical part of the strengthening health system. (A copy of the questionnaire is available from the first author). Descriptive, and exploratory inferential analyses were conducted on the data to examine associations between demographic factors and the probability of medicine shortage.

### Study site

Jumla lies in Karnali Province, the largest and one of the most remote provinces in Nepal. It was selected as it is one of the least accessible districts, situated some 20 h’ drive and over 800 km from the state capital Kathmandu. Jumla consists of one urban municipality and seven rural municipalities with a total population of 108,921 [3].

**Fig. 6:**
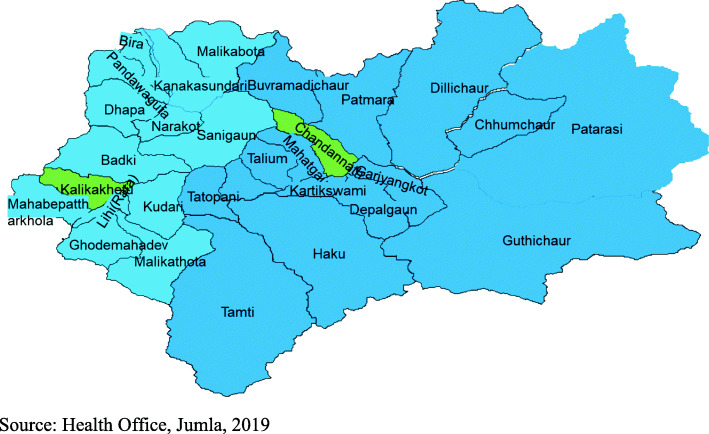
Jumla district map (Green are the referral centres i.e. PHCC- Kalikakhetu and Hospital- Chandannath)

In this district, health facilities comprise 29 Health Posts (HP), a first point of contact for basic health services; a facility one level higher than HP called a Primary Health Care Centre (PHCC); and one hospital, the highest referral centre in the district (Fig. 6). The HP is the first contact point for basic health services. Patients are referred to PHCC and then on to state hospital, provincial hospitals, and, if necessary to specialist tertiary care in central-level hospitals in Kathmandu.

### Recruitment

The Provincial Health Office looks after all the state HFs in the province. Hence they were the first point of contact for seeking access and permission to conduct the survey. Jumla Health Office and as the HFs, were given an information sheet explaining the purpose of the study. The HF information was collected from the person in charge, either at provincial level and/or on-site at the HFs in the same year in early 2019, and were acquired following an identical procedure. Completion of each survey took no more than about 30 min.

### Data analysis

The sample was summarized descriptively. Uncorrected χ^2^ tests for association and uncontrolled logisitic regression analyses were conducted as exploratory procedures to investigate the relationship between demographic factors (gender, years of experience, age and staff qualification) and probability of medicine shortages. All statistical analysis was conducted using IBM SPSS statistical software (Version 26).

### Ethical considerations

Initially ethical approval was granted by the Research Ethics Committee at Liverpool John Moores University and then from the Nepal Health Research Council. A support letter was also obtained from the District Office of Jumla to conduct the study.

## Results

### Health service delivery

The PHCC and hospital both provided Basic Emergency Obstetric and Newborn Care (BEmONC), and of the remining 29 health posts, 23 were classified as birthing centres (BC), the lowest health unit where facility-based births are available. The remaining six health posts were in the process of being upgraded to BC.

Most (*n* = 25) health facilities provided 24/7 delivery and newborn care (birthing centres, PHCC and hospital). All HFs provided ANC, PNC and prevention of mother-to-child transmission (PMTCT) services. The waiting time for all health services at all HF was less than 30 min and all respondents thought that their opening hours were convenient for service users. All HFs kept a register in patient examination rooms for data collection.

Table 1 below summarises the numbers of staff, and rates of any staff shortages, across various posts in the included HFs; with staff numbers and shortages aggregated across all HFs.

**Table 1:** Health workforce in Health Facilities (excluding hospital)

Health staff in 30 HF (excluding hospital)	Number of staff	Staff shortage (%)
Sanctioned Paramedics	88	11.4% gap
Fulfilled Paramedics	78	
Sanctioned Nurses	62	0%
Fulfilled Nurses	62	
Sanctioned other posts	30	10% gap
Fulfilled other posts	27	
Temporary Paramedics (contract)	12	
Temporary Nurses (contract)	31	
Temporary Others (contract)	7	
Volunteer	1	

Table 1 shows all HFs had filled their allocated nurse positions. There was a one-in-nine shortage in paramedic staff, and one-in-ten shortage in auxiliary staff such as laboratory assistants and support staff. However, HFs had contracted temporary staff to cover the unfilled posts or had extra staff available such as nurses. The majority of HFs (*n* = 23) had team meetings once a month, whereas two had none during the past 12 months. All HFs had had meetings with the Health Facility Operation and Management Committee (HFOMC) within the past 12 months. Twenty-four HFs had received supervisory visits in the past 6 months.

### Availability of supplies and equipment

Fifteen HFs considered their facility moderately stocked (with out-of-stock of supplies other than essential medicine), ten considered their facilities were poorly stocked (out-of-stock of supplies including essential medicine) and six were reportedly well stocked (availability of all supplies). The HFs reported as well-stocked were the hospital, the PHCC and four HPs. However, within the past 3 months most facilities (*n* = 21) experienced shortages of essential medicines that are listed on the National List of Essential Medicines Nepal (NLEM) 2016. The most common medicine out of stock was Amoxicillin (*n* = 10), paracetamol (*n* = 10) followed by Vitamin A (*n* = 7) and Metronidazole (*n* = 5). Even though 25 of the facilities were either BEmONC or birthing centres, they experienced a shortage of basic drugs, such as oxytocin, without which it would be difficult to induce and augment labour & prevent postpartum haemorrhage) and magnesium sulphate, which can be used to treat pre-eclampsia and to delay pre-term birth, which are basic requirements for birthing centres. Eight health facilities lacked essential medicine on the day of the study.

### Infrastructure

All health facilities had waste bins, impermeable sharp containers, soap and water, energy infrastructure (solar, generator, grid or direct electricity) and personal mobile phones. However, only fourteen HFs had running water, whilst those without water supply collected water from nearby taps and public water tanks. Seven HFs had telephones, 16 had computer/laptops, 21 had internet connections and 15 had printers on their premises. In terms of facilitating community feedback, only two HFs had a complaint box, both kept outside the HFs building visible for everyone. All HFs had Information Education Communication (IEC) & Behavioural Change Communication (BCC) materials related to maternal and neonatal health available in both Nepali and English languages.

### Maternal and newborn care services

All the birthing centres and BEmONC (25 HFs) used a standard clinical record to monitor events during labour (partograph), birth and postpartum to facilitate written handover. All HFs had policies on the treatment of women and newborns which referred to the Community-Based Integrated Manual of Newborn and Childhood Illness (CB-IMNCI) guidelines by MoHP (2015). All HFs had a system in place whereby mothers of small for dates/premature or sick newborns can be close to and breastfeed their babies.

Five HFs reported that they had to refuse maternal and newborn care at least once due to lack of medicine. Twenty-eight HFs had physical private space for women and their birth companion in the labour area. Some HFs allowed women to have at least one companion of their choice, as culturally appropriate, with them: during labour (*n* = 21), at birth (*n* = 8) and the immediate postnatal period (*n* = 25).

Twenty-five HFs had referred maternal and newborn cases to higher centres for further treatment at some point in time and 22 had done so within the past 12 months. None had received any training related to referral protocols and guidelines within the past 12 months. All HFs that referred maternal and newborn cases had provided referral notes. Twenty health facilities or their communities did not have emergency ambulance transport for women/babies. Four newborns and two postpartum women died before or during transfer to a higher-level facility in the last 12 months, but only one HF had undergone a review of maternal and perinatal deaths.

**Fig. 7:**
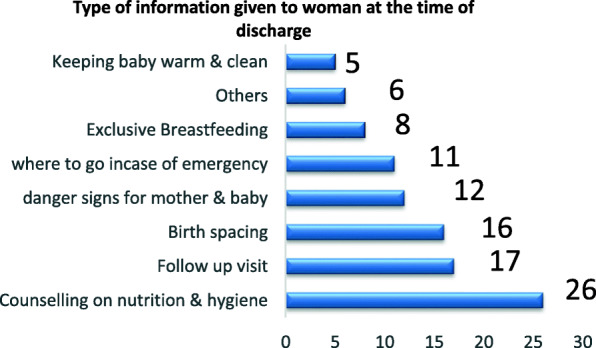
Type of information given to woman at the time of discharge

The most common information given to the women at the time of discharge was counselling on nutrition and hygiene (*n* = 26) and keeping baby warm and clean (*n* = 3) was least reported piece of information in the survey. Other advice covered transport, birth-spacing and immunization (Fig. 7).

Twenty three HFs provided health services free of cost, while the remaining eight HFs said that they provide health services free of cost except for the laboratory services. Of the latter, seven of them had a fee structure displayed on the wall of laboratory (which was not visible to the patient) and one had no fee information on display.

### Exploratory inferential analysis

Uncontrolled logistic regression analysis revealed no evidence that either age, or years of experience of the respondent were significantly associated with the probability of the facility experiencing a shortage of essential medicine within the last 3 months (*p* = 0.741 for age; *p* = 0.156 for years of experience). The two candidate predictors were highly correlated, as expected (*r* = 0.707; *p* < 0.001).

Uncorrected χ^2^ test for associations revealed no evidence that either respondent gender or highest academic qualification (comparing qualified and unqualified staff) was significantly associated with the probability of the facility experiencing a shortage of essential medicine within the last 3 months (*p* = 0.713 for gender; *p* = 0.214 for highest academic qualification). Parameters from all models are summarized in Table 2.

**Table 2:** Parameters from logistic regression analyses and χ^2^ tests for association

Predictor	***p***-value	OR	95% CI for OR	χ^**2**^ statistic (df = 1)
Age	0.917	0.995	(0.913, 1.09)	n/a
Years of experience	0.156	1.07	(0.974, 1.18)	n/a
Gender	0.713	n/a	n/a	0.136
Academic qualification				
Auxiliary nurse (reference)^a^				
Qualified nurse^b^	0.214	n/a	n/a	1.55

^a^Including community medical assistants and auxiliary health workers

^b^Including staff nurses and degree-level educated staff

## Discussion

The study identified that all the birthing centres, PHCC and hospitals provided 24/7 coverage of health services whereas non-birthing centres (HPs) provided services as per office hour opening times. All HFs provided ANC services; the Nepal Health Facility Survey (NHFS) in 2015 also found an equally high proportion (96%) of HFs providing ANC. All the birthing centres, PHCC and hospital provided 24/7 delivery and newborn care. All HFs had basic equipment such as waste bins, sharps containers, soap and water, energy infrastructure (solar, generator, grid or direct electricity) and personal mobile phones. However, only 14 HFs had running water available onsite and the remaining 17 HFs (6 HPs and 11 BCs) collected water from nearby taps and public water tanks. Availability of these basic resources is a pre-requisite for providing quality of care and improving health outcomes for women and newborn [4].

The staff positions were largely filled with 100% staffing rates for nurses, 88% for paramedics and 90% for other staff. HFs also had temporary contract staff to cover some unfulfilled posts. This could be due to the increase in number of the health workforce production in the country as a result of an exponential growth of higher education institutions which offers variety of courses in nursing, medicine and other health sciences. However, the staffing positions in these institutions are based on government allocation (sanctioned positions) not as per the need or demand of local communities/population. Health workforce allocation should be reviewed and based on community need.

This study found that most HFs experienced a lack of stock of essential medicines, which contrasts with Adhikari et al. (2018) who found that PHCCs and HPs in mid-western development region (the equivalent of the current Karnali province) had all essential medicines in stock at that time. However, the limited data currently available precludes the identification of any likely causal factors amongst staff demographics. One explanation for the change in availability of essential medicines could be related to the change in the political system. Before federalisation, the MoHP was responsible for the entire health system of the country, however after federalisation, responsibility has been decentralised to local authorities and perhaps not all local authorities prioritise health [17]. However, the shortage in essential medicine could also be due to an increased demand, limited funds allocated for the medicine supplies or poor logistic management systems [9]. A study conducted by Prinja et al. [14] in 80 public health facilities across 12 districts of Punjab and Haryana states found that the overall availability of medicine was 51.1 and 45.2% respectively. However, in this study they found that more than 90% of the health facilities have had uterotonic drugs (drugs used for induction and augmentation of labour). KC et al. [7] found that the essential medicine and equipment were more available in the higher health facilities compared to the lower ones. It is essential to have basic medicine and equipment available at all times to ensure that better care can be provided to women and newborns. This includes preparedness for complications and emergency situations [7]. Hence, it would be beneficial if the medicine supplies were based on their usage such as looking at patterns of the use and outage over the last few years.

In this study, very few health facilities permitted women to have a partner of their preference present during childbirth. All pregnant women have the right to high quality maternity care which also includes having a companion of their choice during thier care, including labour and childbirth [18, 21]. The support of a companion increases the chance of a spontaneous vaginal birth, shortens labour and decreases caesarean births and other medical interventions [5]. The most common message given to women at the time was counselling on nutrition and hygiene as well as advice on follow-up visits and birth spacing.

Only one of the six maternal and neonatal deaths that took place was reviewed. HFs in Jumla need to conduct reviews of all the maternal and neonatal deaths to understand the possible causes of those deaths. It should also help make service improvements through the formulation and implementation of specific interventions [1]. Twenty-two HFs had referred both mothers and newborns during the past 12 months. However, more than two-thirds did not have any emergency transport services for women or babies.

Service delivery should have well-functioning monitoring and evaluation systems where communities and service users can take part in ensuring the highest quality of care and continuous quality improvement. Hence, there should be a mechanism to receive feedback from service users at the point of delivery. However, in this study only two HFs had a complaint box. Moreover, there was a very limited number of supervisory visits conducted from higher management in the health system to support clinical competence and performance.

### Strengths and weaknesses

One of the strength of this study is that it covered all state HFs in Jumla. The limitations in the study are that it covers only one district in the province, it excludes private health facilities, and that data was largely self-reported by the HF incharge/ acting HF incharge. The number of institutions featured limits the potential for exploratory analysis; furthermore many of the variables collected do not adequately discriminate between institutions and are hence unusable in inferential procedures.

## Conclusions

All birthing centres, PHC and hospitals provide 24/7 delivery and newborn care, and over 90% of HFs had all posts filled, while the rest had temporary contract staff available to cover the unfilled posts. Since these staffing positions are based on government allocation (sanctioned positions), there is an argument to link health worker ratios to the local health needs of the community.

All HFs had policies on treatment of women and newborns. All HFs had a system whereby the mothers of premature or sick newborns can be close to and breastfeed their babies. Most HFs had run out of stock of essential medicine and supplies at some point recently. This suggests that more effective management of medicine in and a better national supplies to health facilities could play an important role in providing quality health services. The patterns of the drug usage over the past few years could help in identifying the demand of the medicine. In addition, better planning and strategic communication between the local, state and federal level policy processes will be necessary to improve health outcomes and meet SDG targets.

Very few HFs permitted women to have a partner of their preference with her during birth. Since only one of the maternal and neonatal deaths were reviewed, there was limited quality control and learning from adverse events. In addition more than two-thirds of HF did not have any emergency transport for women and newborns in case of an emergency, which is an enormous challenge in a remote rural area like Jumla. Having a health workforce is not enough to provide quality services when there is a shortage of supplies. In order to provide quality maternal and neonatal health care, the HF must meet the minimal standards as they are a critical part in the process of strengthening Nepal’s health system.

## Data Availability

The survey questionnaire as well as the datasets used and/or analysed during the current study available from the corresponding author on reasonable request.

## Abbreviations

ANC: Antenatal Care
BC: Birthing Centre
BEmONC: Basic Emergency Obstetrics and Neonatal Care
CEmONC: Comprehensive Emergecny Obstetrics and Neonatal Care
DoHS: Department of Health Service
HF: Health Facility
HP: Health Post
MMR: Maternal Mortality Ratio
MoHP: Ministry of Health and Population
NHFS: Nepal Health Facility Survey
NMR: Neonatal Mortality Rate
PHCC: Primary Health Care Centre
PNC: Postnatal Care
SDG: Sustainable Development Goal
UHC: Universal Health Coverage
UNFPA: United Nations Population Fund
UNICEF: United Nations International Children’s Emergency Fund
WHO: World Health Organization
