# Correction to: Health facility preparedness of maternal and neonatal health services: a survey in Jumla, Nepal

**DOI:** 10.1186/s12913-021-07098-5

**Published:** 2021-10-18

**Authors:** Pasang Tamang, Padam Simkhada, Paul Bissell, Edwin van Teijlingen, Rose Khatri, John Stephenson

**Affiliations:** 1 School of Human and Health Sciences, University of Huddersfield, Huddersfield, UK; 2 Global Health, School of Human and Health Sciences, University of Huddersfield, Huddersfield, UK; 3 Reproductive Health Research, Centre for Midwifery, Maternal & Perinatal Health, Bournemouth University, Poole, UK; 4 Public Health, Liverpool John Moores University, Liverpool, UK; 5 Biomedical Statistics, School of Human and Health Sciences, University of Huddersfield, Huddersfield, UK

**Correction to: BMC Health Serv Res 21, 1023 (2021)**

**https://doi.org/10.1186/s12913-021-07054-3**

Following the publication of the original article [1], the authors identified an error in Fig. 2, the NMR in 2016 should be 21 instead of 15.

**Fig. 2:**
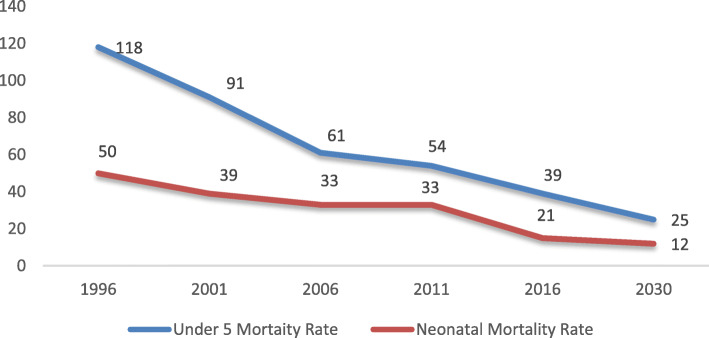
Trends in under − 5 and Neonatal Mortality Rate (Source: NHFS 1991, 2001, NDHS 2006, 2011, 2016)

The correct figure has been included in this correction, and the original article has been corrected.
